# Mobile Technology Adoption in Healthcare—A Behavioral Understanding of Chronic Patients’ Perspective

**DOI:** 10.3390/clinpract15100181

**Published:** 2025-09-28

**Authors:** Andreea Madalina Serban, Elena Druică

**Affiliations:** 1 Faculty of Medicine, Carol Davila University of Medicine and Pharmacy, 050474 Bucharest, Romania; andreea_serban@drd.umfcd.ro; 2 Department of Applied Economics and Quantitative Analysis, University of Bucharest, 030018 Bucharest, Romania

**Keywords:** technology acceptance, perceived risk, mobile health applications, chronic diseases, patient digital self-efficacy

## Abstract

**Background:** In an era of unprecedented technology adoption in healthcare, it is imperative to understand and predict factors influencing users’ perspective. This study employs a risk-integrated technology acceptance model aiming to identify the determinants of the intention to use mobile health applications among patients with chronic diseases in Romania. **Methods:** A face-to-face survey method was used to collect research data from 207 subjects, and the partial least squares structural equation modeling approach was employed for data analysis. **Results:** The behavioral intention to use mobile health applications (INT) was influenced positively by the perceived ease of use (PEOU, f^2^ = 0.358, β = 0.500, *p* < 0.001) and perceived usefulness (PU, f^2^ = 0.271, β = 0.678, *p* < 0.001). Another core predictor, with a negative effect on the intention to use, was the user’s perceived risk of using the technology (RISK, f^2^ = 0.239, β = −0.321, *p* < 0.001), in turn influenced by the perceived degree of cyber-insecurity (CYBER, f^2^ = 0.492, β = 0.639, *p* < 0.001). Digital self-efficacy (DSE) was identified as an external determinant with strong positive influence on PEOU (f^2^ = 0.486, β = 0.610, *p* < 0.001). The model shows strong performance, reflected in a high Tenenhaus goodness-of-fit index (0.770) and solid explanatory power for the outcome variable (adjusted R^2^ = 0.718). **Conclusions:** This study validates an extended risk-integrated technology acceptance model, offering robust insights into the determinants of mobile health application adoption among chronic patients in Romania. The findings provide actionable guidance for designing targeted interventions and healthcare policies to enhance technology adoption in this population.

## 1. Introduction

In the post-pandemic era, the adoption of healthcare technology has accelerated globally. Within the framework of mobile health (mHealth), mobile devices and associated wireless technologies facilitate continuous health monitoring, advanced data analytics, and remote treatment through telemedicine [1,2,3,4]. Despite these advancements in healthcare, the widespread adoption of mHealth solutions remains a significant challenge worldwide. While standing to benefit significantly from mobile technology, patients with chronic conditions in particular encounter substantial barriers, including limited digital literacy, lack of trust in, and concerns about the risks brought by digital solutions. Gaining a deeper understanding of these challenges faced by chronic patients is essential for effectively integrating mobile technology into healthcare delivery in their case, underscoring the need for further research and thoughtful implementation strategies [5].

### 1.1. Knowledge Gap and Research Motivation

While there is a growing body of literature on the adoption of digital solutions in healthcare, the relevance of the findings remains inconclusive. On one side, the large variety of methodological approaches employed in these studies makes comparison and generalizability difficult. On another side, the existing studies do not focus on chronic patients, leaving their needs and drivers of the technology adoption largely underexplored.

One category of limitations of extant research in the field stems from the fact that most of the available studies are mainly descriptive, primarily aiming to map potential factors associated with mHealth adoption [6,7,8,9,10,11]. These studies often rely on qualitative data from focus groups or interviews with patients [8,9,10,11]. Identified facilitators include personal motivation, access to accurate health information, and self-efficacy [10], while reported barriers range from perceived coercion to concerns about restricted autonomy, which may paradoxically lead to patient disengagement [11]. Other descriptive studies propose the theoretical benefits of mHealth for healthcare management [12], some of them subsequently detailing the design of digital solutions intended to deliver these benefits to specific patient groups [13]. However, the absence of a comprehensive theoretical framework in these studies limits their utility for predictive analysis, as well as their capacity to pinpoint the factors that may prove the highest effectiveness in designing and implementing practical interventions.

Another category of existing research focuses on mHealth adoption among the general population, outside the context of chronic diseases. For instance, studies conducted in China and the USA aim at exploring consumer acceptance of mHealth and what shapes the decision-making process [14,15]. The USA study targets a large population of adults (aged 18–87 years) while the Chinese study focuses on young adults (aged 18–30). No other inclusion criteria were applied to the participants. The same general target population is involved in a study that examines mHealth applications aimed at promoting mindfulness to improve general health outcomes [16]. Along the same lines, other studies focused on acute healthcare challenges scenarios, such as the SARS-CoV infections [17,18,19] or technology adoption among rural populations [20]. The adoption of advanced technologies, specifically home care robots, was investigated in a study on home healthcare in the United States, encompassing perspectives from both patients and healthcare providers [21]. Two studies performed in China evaluated determinants of healthcare technology acceptance in the general population [22], as well as among elderly individuals [23]. The characteristics of different populations targeted by studies on technology adoption in health are illustrated in Table 1.

Generalizing findings from such studies to patients with chronic conditions disregards their distinct challenges and can result in misleading assumptions that foster ineffective, or even detrimental, interventions.

Our study seeks to overcome the main limitations of the extant literature. In addition to focusing on the underexplored population of patients with chronic diseases, we employ an empirical approach based on an extended version of the Risk-Integrated Technology Adoption Model (RITAM).

### 1.2. Theoretical Background

Over the past decades, various theories and their extensions have been applied to explain technology acceptance across different domains. Each framework identifies distinct factors influencing users’ attitudes toward a specific technology, as well as their intention to adopt and ultimately use that solution. Some of the most important theories include: the Theory of Reasoned Action, the Technology Adoption Model, the Unified Theory of Acceptance and Use of Technology, the Social Cognitive Theory, The Theory of Interpersonal Behavior, The Theory of Planned Behavior, The Perceived Characteristics of Innovating Theory, The Model of PC Utilization, The Motivational Model, and the Innovation Diffusion Theory [24,25,26,27].

Among them, a central role is played by the Technology Adoption Model (TAM henceforth). This theory is considered the gold standard for technology adoption in many fields; it is therefore unsurprising that this concept has also been widely applied to explain the adoption of digital healthcare tools [10]. TAM was initially developed by Fred Davis in 1985 and is centered around two theoretical constructs, namely perceived usefulness (PU henceforth) and perceived ease of use (PEOU henceforth) [27]. For the purpose of his initial study, Davis defined PU as “the degree to which a person believes that using a particular system would enhance his or her job performance”. In this context, a system high in PU became one in which the user acknowledged a use-performance contingency. At the same time, Davis characterized PEOU as “the degree to which a person believes that using a particular system would be free of effort”. In the described model, an application perceived as being easy to use was very likely to be chosen by users over alternative options when all other conditions are considered equal [28].

Since its introduction in 1989, TAM has been widely applied across various contexts and further refined to explore potential determinants of PEOU and PU in greater detail. A significant theoretical advancement in the field of technology adoption is the incorporation of perceived risk as a key determinant of behavioral intention. In 2014, in a study evaluating smart grid consumer engagement, the authors highlighted the importance of considering risk as a subjective factor within TAM, leading to the development of the Risk-Integrated Technology Adoption Model (RITAM henceforth) [29]. Given the conflicting findings in previous studies concerning the relationship between perceived risk and perceived benefit (usefulness), RITAM proposes hypotheses that suggest mutually negative interactions between these two determinants of behavioral intention [29]. While the term RITAM was coined in 2014 [29], the idea of risk influence on attitudes was not new. Prior research had already explored the impact of perceived risk on user behavior in fields such as e-commerce adoption [30], nanotechnology [31] or online banking [19].

When analyzing determinants of technology adoption in different areas of healthcare, several theoretical TAM extensions have been evaluated. Some authors analyzed the adoption of drones in the context of COVID-19 pandemics in Israel [7]. The authors proposed and tested an integrated model that combines the Technology Acceptance Model (TAM), Task-Technology Fit (TTF), social motivation, and drone-related perceived risks to examine the intention to use drones in public health emergencies. Their findings indicate that the intention to use drones is significantly influenced by perceived ease of use, behavioral attitudes, individual-technology fit, task-technology fit, and social recognition. Notably, their study found that perceived ease of use did not predict perceived usefulness, a result the authors attributed to the unique context of remote drone operation [7].

Another noteworthy study aimed to identify the determinants of telemedicine adoption within the rural population of Pakistan [20]. The authors proposed an extended TAM framework that incorporated additional factors such as resistance—defined as social inertia in the face of change; trust—the confidence users have in a solution and its ability to deliver as promised; facilitating conditions—the availability of organizational and technological infrastructure; technological anxiety—the fear or apprehension towards new digital technologies; and perceived risk and privacy concerns. Their findings revealed that all these factors, except privacy concerns, significantly influenced the intention to adopt telemedicine services [20].

In another study, the authors sought to identify the determinants of healthcare home robot adoption [21]. Conducted across more than 50 cities in the United States, the study included a sample of 108 participants, comprising both patients requiring home healthcare services and practitioners delivering those services. The conceptual model integrated factors such as performance expectancy; effort expectancy; social influence—defined as the degree to which stakeholders perceive that important others believe they should use a specific technology; facilitating conditions; trust—the belief that healthcare robots have the capability and integrity to protect personal information; and privacy, ethical, and legal concerns. The study found that social influence, facilitating conditions, and trust positively impacted the intention to use healthcare home robots. Conversely, privacy and ethical concerns negatively affected the intention to adopt this technology, while the influence of legal concerns did not prove to be significant [21].

Another notable study aimed to identify the factors influencing patients’ acceptance of mobile medical platforms [22]. The authors integrated the Theory of Planned Behavior (TPB), the Technology Acceptance Model (TAM), and three patient-centered factors: perceived convenience, perceived credibility, and perceived privacy risk. The findings revealed that all the traditional determinants from TPB and TAM significantly impacted behavioral intention. However, an interesting outcome was that social influence did not have a significant effect on the intention to use mobile medical platforms [22].

An essential aspect of technology acceptance in healthcare is the adoption of telemedicine services, particularly in the context of the COVID-19 pandemic and the significant post-pandemic shifts in how telemedicine is perceived. Telemedicine adoption was the primary focus of a study which evaluated the factors predicting ophthalmology patients’ intention to use telemedicine during the pandemic [18]. Notably, the authors chose to exclude social influences from their model, as previous research suggested that these factors have little impact in voluntary contexts. Instead, their model incorporated the classic TAM determinants along with facilitating conditions, such as internet access, relevant equipment, and the ability to effectively use technology [18].

Along similar lines, a study examining the adoption of telemedicine applications in Indonesia during the pandemic era employed an original model comprising five constructs: convenience, health stress, perceived usefulness, satisfaction, and continuance intention [19]. Notably, the findings revealed that perceived usefulness did not have a statistically significant impact on users’ intention to continue using mobile health technology, a result that contrasts with common assumptions in technology adoption literature [19]. These outcomes highlight the complexity of factors driving continued engagement with mobile health technologies and underscore the importance of context-specific research.

Complementing prior research, our study makes a substantial contribution to the field by introducing two key areas of innovation. Firstly, it extends the existing theoretical framework through the use of a RITAM-based approach to the context of technology adoption among patients with chronic diseases. This application provides new insights into the factors influencing patient engagement with health technologies, thereby advancing the understanding of technology acceptance in healthcare settings. Despite the various adaptations employed by different authors in evaluating technology acceptance in healthcare, TAM has consistently proven to be an effective framework for understanding this phenomenon. However, research assessing risk as a potential determinant of behavioral intention remains limited; we contend that its inclusion is particularly relevant when evaluating mHealth adoption by patients with chronic diseases, given the vulnerable nature of technology users and the sensitive nature of digital data exchange.

Secondly, in addition to RITAM determinants, our study follows an innovative approach, by incorporating digital literacy as a particular expression, relevant to our population, of the facilitating conditions present in several other studies in the field. The term digital literacy was first used in 1997 [32] and it was defined as: “A set of skills to access the internet, find, manage and edit digital information; to participate in communication and interact with an online network of information and users. Digital literacy is the ability to properly use and assess digital resources, tools and services and apply them to lifelong learning processes” [32].

Several studies have explored the relationship between digital skills and the use of healthcare technology. In the specific context of patients with chronic conditions, those who use digital health tools less frequently tend to have lower health-related technology literacy, express greater concerns about the security of their personal data, and perceive these tools as less useful [7]. In a similar vein, other authors examined the use of a mobile app for self-management in patients with Type 2 diabetes [33]. Although the app was shown to lower hemoglobin A1c (HbA1c) levels, indicating improved health outcomes, its adoption rate among the target population was only 18.2%. Older patients, those with lower individual motivation, and those with lower perceived usefulness of digital health tools were less likely to engage with the app [33].

## 2. Materials and Methods

### 2.1. Conceptual Model

The disconnect between the proven clinical benefits of healthcare technology and its low adoption rates underscores the need to address the behavioral factors involved. To bridge this gap and enhance the success of digital health interventions, it is essential to devote more research to understanding the drivers of technology adoption in healthcare. Our main research objective is to contribute to this critical research area by offering insights into the behavioral factors that influence the acceptance and use of mHealth technologies among patients with chronic conditions.

To achieve this objective, we utilize RITAM as the theoretical framework for developing our conceptual model. Within the scope of this study, PEOU denotes the belief that mobile health applications require minimal effort to operate, while PU reflects the belief that such applications can improve relevant aspects of health. The additional component, RISK, addresses patients’ concerns about mobile health technology, including apprehensions about the security and transmission of sensitive data and the potential for communication errors leading to medical mistakes.

In addition to RITAM determinants, our study follows an innovative approach, by incorporating digital literacy as an external factor influencing PEOU [34,35].

Our first research question (RQ) is:

*RQ1: How do perceived usefulness (PU), perceived ease of use (PEOU) and perceived risk (RISK) influence the behavioral intention to use mobile health apps among patients with chronic diseases?*

Digital self-efficacy (DSE) is recognized as a crucial determinant of digital technology use [34]. Research shows that both actual digital skills and individuals’ beliefs in their abilities—particularly DSE—significantly impact the effective use of digital systems. While digital competences and confidence in those competences are related, they independently affect learning, motivation, and performance. Digital self-efficacy is defined as one’s confidence regarding the use of a digital systems (e.g., internet, computer, web applications, etc.). Various scales have been proposed to measure DSE, with one of the most widely adopted being the scale developed in 2022 [35]. This scale is grounded in Bandura’s concept of self-efficacy from Social Cognitive Theory, which defines self-efficacy as an individual’s confidence in their ability to successfully execute a specific task [36]. The DSE scale is also informed by the European Commission’s Digital Competence Framework for Citizens, DigComp 2.1 [37]. Using this scale, we aim to address the research question:

*RQ2: How does digital self-efficacy (DSE) influence the perceived ease of use (PEOU) of mobile health apps among patients with chronic diseases?*

Several studies indicate that security concerns are a primary barrier to technology adoption, particularly when sensitive data is involved [38,39,40,41]. For the purpose of our study, cyber insecurity (CYBER) will be defined as a patient’s reluctance to adopt mobile technology due to concerns regarding adverse conditions, stresses, attacks, or compromises on systems that include cyber resources [42]. In alignment with previously validated RITAMs, we will address the following question:

*RQ3: How does the perceived cyber insecurity (CYBER) influence the perceived risk (RISK) of using mobile health apps among patients with chronic diseases?*

**Figure 1:**
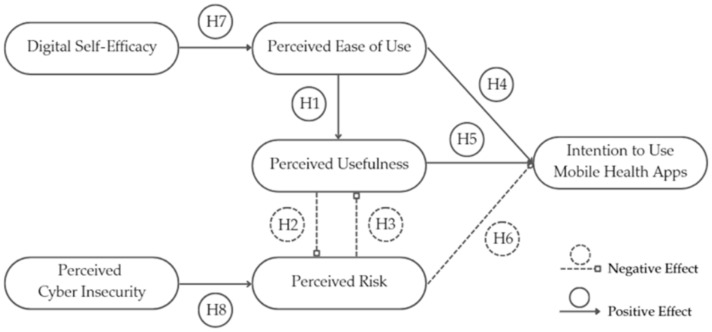
Extended Risk-Integrated Technology Adoption Conceptual Model. Perceived ease of use (PEOU), perceived usefulness (PU), and perceived risk (RISK) are the main determinants of the behavioral intention to use mHealth among patients with chronic conditions (INT). The measurement model includes digital self-efficacy (DSE) as a determinant of PEOU, as well as cyber insecurity (CYBER) as a determinant of RISK.

To address our research questions, we have developed several hypotheses, in alignment with the RITAM framework extensively validated in the literature in relationship with other technology adoption applications [43,44]. To better illustrate the research hypotheses, as well as the interactions among the analyzed components, we have created a graphical representation of the conceptual model (Figure 1).

The determinants influencing the intention to use mobile health apps will be evaluated through hypotheses H1–H6. Consistent with the foundational TAM framework, we will first assess the positive correlation between the perceived ease of use of mobile health apps and the perception of their usefulness in managing chronic disease care [29,30,45]. The first hypothesis is:

**H1:**  *Perceived ease of use (PEOU) is positively related to perceived usefulness (PU).*

The following two hypotheses will investigate the relationship between the perceived usefulness of a mobile health app and the perceived risks associated with using such digital tools [43]. We will explore this relationship bidirectionally, as part of the second and third hypotheses:

**H2:**  *Perceived usefulness (PU) is negatively related to Perceived Risk (RISK).*

**H3:**  *Perceived Risk (RISK) is negatively related to Perceived usefulness (PU).*

Subsequently, we will assess the potential determinants of the intention to use a mobile health app, in line with the RITAM framework [43,44,46]. Specifically, we will analyze the relationships between PEOU and PU with INT, hypothesizing a positive effect, as well as between RISK and INT, hypothesizing a negative effect, as evaluated through:

**H4:**  *Perceived ease of use (PEOU) is positively related to intention (INT).*

**H5:**  *Perceived usefulness (PU) is positively related to intention (INT).*

**H6:**  *Perceived risk (RISK) is negatively related to intention (INT).*

Drawing on the literature on digital self-efficacy, we will test the hypothesis that DSE positively influences PEOU [34,47]. Specifically, we anticipate that a patient’s self-assessed level of digital readiness will be positively correlated with their perception of minimal effort required to use a mobile health app. The relationship between DSE and PEOU will be evaluated through the following hypothesis:

**H7:**  *Digital Self-Efficacy (DSE) is positively related with perceived ease of use (PEOU).*

Among the exogenous variables with significant influence on perceived risk in the original RITAM, we test the Perceived Cyber Security Threads (Cyber Insecurity—CYBER) [43]. Previous research conducted on the Romanian population has demonstrated that perceived cyber insecurity significantly predicts perceived risk in the adoption of new technologies [44]. In the context of mHealth applications, a substantial body of literature highlights critical concerns related to privacy and security within digital environments. These concerns primarily focus on the protection of sensitive patient information, ensuring confidentiality, and safeguarding against unauthorized access. Furthermore, the safety of data exchanges between patients and healthcare providers is emphasized, as secure communication channels are essential for maintaining trust and compliance with legal regulations on data protection [19,48,49]. The hypothesis developed to test the relationship between CYBER and RISK in our study is:

**H8:**  *Perceived Cyber Insecurity (CYBER) is positively related to the perceived risk (RISK).*

### 2.2. Respondent Selection and Data Collection

This study was conducted in the Internal Medicine Department of Colentina Medical Hospital in Bucharest. As a tertiary-level healthcare facility, the hospital treats patients from various regions across Romania, referred from secondary or primary care centers. After receiving ethical approval from the Institutional Review Board, we used non-probability convenience sampling to select our sample, administering the questionnaire in-person to patients hospitalized in the department over a 30-day period. The inclusion criteria were a confirmed diagnosis of a chronic disease made at least three months prior to the study, as well as the absence of any physical or psychological disability that would impede participation, as assessed by medical records and clinical evaluation.

For this study, we defined chronic disease according to the definition employed by the Centers for Disease Control and Prevention: a condition lasting one year or more that requires ongoing medical care, limits daily activities, or both [50]. All questionnaires were administered by a single investigator following a standardized protocol. After explaining the study’s purpose and inviting patients to participate, informed consent was obtained in writing, in accordance with institutional ethical guidelines. No financial incentives were offered for participation. Based on the inclusion criteria, 241 patients were identified, of whom 85.9% (n = 207) agreed to participate in the study.

### 2.3. Measurement

The questionnaire employed a 7-point Likert scale to capture responses. It included both positively and negatively worded statements, along with reverse-scored items, to minimize the effects of acquiescence bias. The latent constructs, survey items, and corresponding bibliographical references are detailed in Table S1 in the Supplemental Materials.

### 2.4. Statistical Analysis

To analyze our data, we employed Partial Least Squares Structural Equation Modeling (PLS-SEM), due to its ability to estimate complex models using relatively small sample sizes, and its flexibility in accommodating various types of data distributions [51]. PLS-SEM is an iterative algorithm that consists of two models: the measurement model and the structural model. In the measurement model, PLS regression analysis calculates weights and loadings to assess the contribution of each observed variable to its corresponding latent construct. For each construct, a single score is computed for each observation, serving as a proxy for the theoretical concept. This step is crucial for determining how well the constructs represent the underlying theoretical framework.

The second component of PLS-SEM is path analysis, which examines the direct and indirect relationships among the constructs within the model. By integrating these components, PLS-SEM not only enhances the robustness of the analysis but also provides valuable insights into the structural relationships between variables [52]. Unlike its Confirmatory Factor Analysis counterpart, PLS-SEM is a predictive method, often used to inform practical interventions, one of the many reasons why we found it relevant in our case. Our analysis was conducted using WarpPLS^®^ 8.0 and SmartPLS^®^ 4 software.

## 3. Results

### 3.1. Demographics

Our sample consisted of 207 participants, offering a relatively balanced representation across age groups. Participants ranged from 22 to 92 years old, with an average age of 57.8 years and a median age of 59. Their demographic characteristics are summarized in Table 2.

### 3.2. The Measurement Model

In order to confirm the reliability of our measurements, we evaluated the composite reliability and discriminant validity. The composite reliability values ranged from 0.901 for RISK to 0.991 for INT. Additionally, all Cronbach’s alpha values exceeded the recommended threshold of 0.7, varying from 0.836 for DSE to 0.982 for PU [53]. The lowest average variance extracted is 0.694 for RISK, above the recommended threshold of 0.5 [54]. To assess discriminant validity, we examined the correlations among the latent variables and compared them with the square roots of the average variance extracted (AVE). The off-diagonal correlation values, below 0.8, confirmed the discriminant validity of the constructs [55]. The next step was to evaluate the heterotrait-monotrait (HTMT) ratios, in order to further assess discriminant validity of our measurement instrument. Good values for the HTMT ratios are considered the ones below 0.9, and ideal ones are below 0.85 [56]. Additionally. SRMR (Standardized Root Mean Square Residual) was 0.063, below the recommended threshold of 0.08, indicating good model fit [57]. Detailed reliability measurements are presented in Table S2 in the Supplemental Materials. These results indicate excellent internal consistency and support the reliability of the outcomes.

### 3.3. Determinants of the RITAM Predictors

Table 3 presents the estimated coefficients of two submodels depicted in Figure 1—one that predicts PEOU, and another one that explains RISK—along with the effect sizes of each predictor. Both H7 and H8 were confirmed. The effect sizes for these determinants were substantial, with f^2^ = 0.486 for the impact of DSE on PEOU (*p* < 0.001) and f^2^ = 0.448 for the impact of CYBER on RISK (*p* < 0.001), highlighting them as effective intervention targets. The models also demonstrated excellent Tenenhaus goodness-of-fit indices—0.817 for the PEOU model and 0.703 for the RISK model—with strong explanatory power at 68.7% and 53.2%, respectively. Among the demographic variables, age was the only factor significantly associated with both PEOU and RISK (f^2^ = 0.126, *p* < 0.001 for the effect on PEOU, f^2^ = 0.055, *p* < 0.05). Younger patients reported higher perceived ease of use, while older patients perceived greater risks associated with mobile health technology use.

### 3.4. The Structural Model

In line with the RITAM framework described in the literature, first we report the model that assesses the path proposed in H2, evaluating the effects of perceived usefulness on perceived risk, while considering the influence of perceived ease of use on perceived usefulness as indicated in H1. Both hypotheses are supported, with the model showing strong performance, evaluated through the model fit and quality indices. In our results, this was reflected in a high Tenenhaus goodness-of-fit index (0.742) and solid explanatory power for the outcome variable. The estimated coefficients of this model are reported in Table 4 (A—Estimated Coefficients, B—Effect Sizes), confirming also hypotheses H4–H6.

For the subsequent step in our analysis, we conducted a diagnostic check using Warp2 bivariate causal direction ratios and differences to evaluate whether there is support for reversing the link between PU and RISK, in line with the H1–H3 pathway. The results do not offer evidence to support a reversed relationship as proposed by H3 (Supplementary Materials, Table S3).

## 4. Discussion

Romania provides a particularly instructive context for examining mHealth adoption, as it combines relatively high technology penetration [58] with a severe shortage of healthcare professionals [59]. The adoption of mHealth solutions is significantly hindered by disparities in digital resources and literacy between individuals residing in urban versus rural regions. Suggested barriers to digital health adoption are the inadequate infrastructure, limited financial resources, and a lack of comprehensive policies supporting digital health initiatives [60].

Our research employs a rigorous methodology to identify the main determinants of mHealth adoption among Romanian patients with chronic conditions. To reach this goal, we have designed and tested an extended conceptual model based on the RITAM theoretical framework, with the addition of DSE as a predictor of PEOU. In line with standard practice in this research field, intention was assessed through self-reported items adapted from validated RITAM domains and complemented with measures of digital self-efficacy derived from the European Commission’s Digital Competence Framework for Citizens. Content validity of the adapted outcome was confirmed through expert review, in order to ensure clarity, comprehensibility, and relevance for the target population. Previous research has consistently demonstrated that intention to use technology serves as a robust proxy for actual technology adoption [61,62,63], further supporting the validity of the outcome measures in this research.

Our results corroborate established findings in the literature. Firstly, numerous healthcare technology adoption frameworks have identified PEOU as a key determinant of usage intention, a relationship that our study also confirms [15,17,20]. Secondly, research based on TAM consistently evaluates the impact of PU on the intention to adopt mobile health technologies. In line with prior studies across various healthcare technology applications, our research demonstrates a positive effect of PU on the intention to use these technologies [18,21,23]. Lastly, RISK, a central element of RITAM-based models, is supported by our findings, which reveal a statistically significant negative effect of perceived risk on the intention to use mobile health applications.

Interestingly, our analysis does not indicate a bidirectional relationship between RISK and PU. Our evaluation using Warp2 bivariate causal direction ratios provides rather solid evidence supporting a unidirectional connection; still, a complex interplay in which perceptions of usefulness and risk are interdependent should be investigated in future research, as users’ perceptions of a technology’s utility can impact their evaluation of associated risks, and vice versa. Specifically, an increased perception of a mobile health app’s usefulness may reduce perceived risk due to greater confidence in the app’s benefits. Conversely, elevated perceived risk can lead to a diminished perception of the app’s usefulness, as users may grow more doubtful about its value. We emphasize the need to address both PU and RISK within technology adoption models, especially in healthcare contexts where these factors are crucial in shaping user acceptance. Future research should incorporate these potential bidirectional influences to enhance the design and effectiveness of interventions aimed at improving technology adoption among patients with chronic diseases.

Our study brings several contributions to this field of research. Firstly, it advances the understanding of the determinants of mobile health technology adoption specifically within the vulnerable population of adult patients with chronic diseases. While RITAM has been extensively applied across various domains, our research introduces an innovative approach by validating an extended version of this model within the healthcare context, with DSE and CYBER as a key added component. In the context of rapidly advancing healthcare technology integration, this study provides a clear image on the statistically significant relationships in the conceptual model and the respective effect sizes. Thus, it manages to provide actionable insights into policy design and implementation strategies tailored to the needs of chronic patients, who stand to benefit most from such advancements.

Moreover, our study not only confirms the applicability of the RITAM framework within the healthcare context but also extends the theoretical model by incorporating two additional determinants: DSE, influencing PEOU, and CYBER, affecting RISK. Both constructs demonstrated statistically significant impacts on their respective RITAM predictors, offering valuable insights for various stakeholders. Healthcare professionals, in particular, should consider these findings when recommending mobile health solutions to patients. For instance, as telemedicine becomes increasingly prevalent and integrated into standard care across various medical specialties, it is imperative to recognize that patients may require targeted training to achieve a sufficient level of DSE. Moreover, our results highlight the importance of clear communication regarding data privacy and cybersecurity when engaging with patients, ensuring they fully understand the risks and safeguards associated with these digital health tools.

Another key group that stands to benefit from our study’s findings is healthcare technology designers. Consistent with prior research recommendations [17], our results underscore the importance of intuitive design and a strong emphasis on usability. To enhance inclusiveness, it is advisable for technology experts to design mobile applications that closely mimic key design elements of widely adopted platforms, such as WhatsApp^®^ for telemedicine chat services, Zoom^®^ for video consultations, or Dropbox^®^ for file sharing. This approach can facilitate user adoption by leveraging familiar interfaces and functionalities, thereby improving the overall user experience and effectiveness of mobile health technologies.

The findings of this study also carry significant implications for healthcare policymaking, with Romania serving as a pertinent case study that mirrors challenges faced by healthcare systems globally. The incidence of chronic diseases in Romania has been steadily increasing over the past decades [64], leading to a growing number of patients in need of long-term medical care. Concurrently, many regions in the country continue to grapple with critical shortages of medical personnel. In this context, healthcare technology presents proven benefits in addressing these challenges and, if implemented effectively, could have a profound impact on the lives of millions of patients with chronic conditions. Our study offers valuable insights that can guide the formulation of policies aimed at addressing the actual determinants of technology adoption, ensuring that such initiatives are both practical and attuned to the needs of the population.

While our study offers valuable information, it is not without limitations that warrant consideration. Firstly, the study’s design introduces a sampling bias due to the use of convenience sampling. All eligible patients admitted for hospital care were invited to participate, which precluded the possibility of randomization. Additionally, the study was conducted at a single center, Colentina Hospital in Bucharest. Although this hospital serves a broad national population across various chronic diseases, the Internal Medicine department has a strong focus on rheumatological conditions. This specialization is reflected in the disease types prevalent among our participants and the gender distribution in our sample. The higher prevalence of autoimmune and rheumatological diseases among females, compared to males, is mirrored in a slight gender imbalance in our study. Future research should aim to replicate and extend these findings across different segments of the chronic patient population to enhance generalizability.

Another significant limitation of our study is inherent to its design. The decision to administer the questionnaire through face-to-face interactions with the researcher introduces the potential for social desirability bias, which may affect the validity of the responses. To mitigate this, we emphasized the voluntary and anonymous nature of participation. This approach was intentionally selected to enhance inclusiveness, ensuring that participation was accessible even to those who might face challenges in completing a digital form. Considering the vulnerable nature of our study population—adult patients with chronic diseases—we prioritized minimizing any technology-related barriers to participation.

To enhance the generalizability of our findings in future studies, several methodological approaches could be employed. Prioritizing multi-center recruitment could allow researchers to capture a broader range of experiences and perspectives, strengthening the representativeness of the sample. Implementing a randomized sampling approach could also be crucial, as it minimizes selection bias and ensures a more diverse participant pool. In addition, employing stratified sampling based on the type of chronic disease could further diversify the patient population, allowing for comparisons across different conditions. To mitigate social desirability bias, one potential strategy could involve administering surveys through individuals not directly associated with the medical team, thereby fostering a sense of anonymity and encouraging more honest responses. Conducting these interactions outside of patients’ admission periods might also help to alleviate any pressure felt during in-person interviews. Alternatively, in settings where access to technology is not a barrier, online surveys should be considered a viable method for data collection, as a valid way to mitigate social desirability bias. Incorporating a mixed-methods approach, such as combining qualitative interviews with quantitative surveys, could provide deeper insights, particularly for patients with lower levels of technology use. This would help identify specific barriers they face in accessing digital health solutions and contribute to more tailored interventions in future research.

Moreover, future research could leverage modern machine learning (ML) techniques, to explore complex, nonlinear relationships between determinants of mobile health adoption. Evidence from other domains shows that sequential and nonlinear effects can exist among predictors, as observed in large-scale studies on quality of life, stress, and sleep patterns in regionally diverse populations. Such dual approaches, combining conventional regression and mediation analyses with ML approaches, enhance the robustness of findings. In particular, the identification of threshold and plateau effects highlights how ML techniques can uncover nuanced dynamics in psychosocial processes [65]. Applied to chronic patient populations, these approaches may uncover complex interactions among clinical, behavioral, and technological factors, informing the design of more tailored interventions.

Despite the limitations discussed, our study offers substantial value and makes important contributions to the understanding of mobile health technology adoption among chronic patients. While the convenience sampling and single-center design may introduce some biases, these factors do not diminish the relevance or potential impact of our findings. Indeed, the insights gained from this research are crucial for healthcare professionals, technology designers, and policymakers alike.

## 5. Conclusions

In the context of rapidly expanding technological advancements in healthcare, understanding and anticipating the factors that shape users’ perspectives is crucial. This research leverages an adapted RITAM framework to explore the key drivers behind patients’ intentions to use mHealth solutions, focusing specifically on individuals with chronic illnesses in Romania. Using PLS-SEM, our study reveals that behavioral intention to adopt mHealth applications is positively influenced by PEOU and PU. Conversely, RISK, shaped by concerns over cybersecurity, emerged as a significant factor negatively affecting adoption intentions. Additionally, DSE was identified as an external variable with a strong positive impact on PEOU.

To further solidify and expand upon our results, larger-scale studies across diverse segments of the chronic patient population are warranted. Complementarily, ML techniques could be applied to reveal additional insights into technology adoption behaviors. This study provides a strong foundation, offering valuable guidance for the development of more effective and inclusive mobile health interventions, ultimately leading to improved health outcomes and enhanced patient engagement.

## Figures and Tables

**Table 1:** Literature Overview—Population Characteristics in Studies evaluating Technology Adoption in Healthcare.

Geographical Coverage	Age Groups	Context	Reference
USA	18–87	Measuring the influence of design simplicity on mHealth adoption among existing general patient portal users	[16]
China	18–30	Understanding consumer’s acceptance of health portals among young adults from the general population	[15]
USA	18–29	Interviews evaluating perceptions on mHealth mindfulness-based interventions in promoting better health outcomes	[16]
Israel	≥18	Exploring the intention to use technology in public health emergencies (i.e., COVID-19 pandemics)	[17]
Greece	≥18	Identifying determinants of teleophthalmology usage during the COVID-19 pandemics among patients with unspecified ophthalmologic concerns	[18]
Indonesia	≥18	Evaluating determinants of mobile health application use among the general population during the COVID-19 pandemics	[19]
Pakistan	20–50	Evaluating telemedicine adoption among the rural population	[20]
USA	≥18	Identifying determinants of the adoption of home healthcare robots for general home healthcare beneficiaries	[21]
China	All ages included (n1 = 27 individuals <18-year-old, n2 = 362 subjects ≥18-year-old)	Evaluating Mobile Medical Platform adoption among the general population	[22]
China	>60	Exploring determinants of technology adoption among elderly individuals; general references to healthcare technology	[23]

**Table 2:** Characteristics of Study Participants.

Characteristics	Category	Frequency (n)	Percentage (%)
Age (years)	18–30	6	2.8
	31–45	37	17.9
	46–60	69	33.5
	>60	95	45.9
Gender	Female	142	68.6
	Male	65	31.4
Education	Middle education or less	134	64.7
	Higher education	73	35.3
Area of Residence	Urban	128	61.8
	Rural	79	38.2
Income Level *	<2000 RON (approx. 400 EUR)	83	40
	2001–4000 RON (approx. 400-800 EUR)	70	33.8
	4001–6000 RON (approx. 800–1200 EUR)	27	13
	6001–8000 RON (approx. 1200–1600 EUR)	12	5.8
	8001–10,000 RON (approx. 1600–2000 EUR)	7	3.4
	>10,000 RON (approx. 2000 EUR)	8	3.9

* 1 RON = 0.2 EUR.

**Table 3:** The Measurement Model—Latent Variables Coefficients.

	Perceived Ease of Use (PEOU)		Perceived Risk (RISK)	
	Estimated Coefficients	Effect Sizes	Estimated Coefficients	Effect Sizes
**Digital Self-Efficacy (DSE)**	0.610 ***	0.486	-	-
**Perceived Cyber Insecurity (CYBER)**	-	-	0.639 ***	0.448
**Age (AGE)**	−0.213 ***	0.126	0.156 **	0.055
**Education (EDUC)**	0.079	0.04	−0.057	0.014
**Gender (GENDER)**	0.063	0.003	0.006	0.000
**Area of Residence (RESID)**	0.006	0.001	−0.015	0.000
**Income Level (INCOME)**	0.072	0.034	−0.049	0.015
**R^2^/Adjusted R^2^**	68.7%/67.8%		53.2%/51.8%	
**Tenenhaus GoF**	0.817 (large)		0.703 (large)	

—*p* < 0.1; *—*p* < 0.05; **—*p* < 0.01; ***—*p* < 0.001.

**Table 4:** RITAM H1–H2 Pathway.

A. Estimated Coefficients						
	Direct Effects			Indirect Effects		Total Effects
	Usefulness	Risk	Intention	Risk	Intention	Intention
**Perceived ease of use**	0.700 ***	-	0.256 ***	−0.502 ***	0.244 ***	0.500 ***
**Perceived usefulness**	-	−0.717 ***	0.348 ***	-	0.230 ***	0.678 ***
**Perceived Risk**	-	-	−0.321 ***	-	-	−0.321 ***
**Age**	-	-	−0.011	-	-	−0.011
**Gender**	-	-	−0.021	-	-	0.021
**Education**	-	-	−0.054	-	-	−0.054
**Area of residence**	-	-	−0.005	-	-	−0.005
**Income Level**	-	-	0.118 *	-	-	0.118 *
**R^2^/Adjusted R^2^**	49.0%/48.7%	51.4%/51.2%	72.9%/71.8%			
**Tenenhaus GoF**	0.770 (large)					
**B. Effect Sizes**						
	**Ease of Use**		**Usefulness**	**Risk**		**Intention**
**Perceived Ease of Use**						0.358
**Perceived Usefulness**			0.490			0.271
**Perceived Risk**						0.239
**Cyber Insecurity**				0.492		0.137
**Digital Self-Efficacy**	0.633		0.297			0.247
**Age**						0.005
**Gender**						0.001
**Education**						0.019
**Area of residence**						0.000
**Income Level**						0.048

—*p* < 0.1; *—*p* < 0.05; **—*p* < 0.01; ***—*p* < 0.001.

## Data Availability

Data will be made available on request.

## Supplementary Materials

The following supporting information can be downloaded at: https://www.mdpi.com/article/10.3390/clinpract15100181/s1, Table S1. Latent Constructs and Survey Items of Study Questionnaire, Table S2. Measurement Reliability Assessment, Table S3. Causality Assessment Coefficients Analysis.

## Abbreviations

The following abbreviations are used in this manuscript:

INT	Intention to use a mobile health application
PEOU	Perceived Ease of Use
PU	Perceived Usefulness
RISK	Perceived Risk of using a mobile health application
CYBER	Perceived degree of Cyber-insecurity
DSE	Digital Self Efficacy
mHealth	Mobile Health
RITAM	Risk-Integrated Technology Adoption Model
TAM	Technology Adoption Model
CDC	Centers for Disease Control and Prevention
PLS-SEM	Partial Least Square Structural Equation Modeling
AVE	Average Variance Extracted
SRMR	Standardized Root Mean Square Residual
HTMR	Heterotrait-Monotrait Ratios
ML	Machine Learning
