# Supplementary material for: Mobile Technology Adoption in Healthcare—A Behavioral Understanding of Chronic Patients’ Perspective
Source: Clin Pract. 2025 Sep 28;15(10):181. doi: 10.3390/clinpract15100181 (PMC12562515; doi:10.3390/clinpract15100181)
Supplement: Supplementary file 1 [file clinpract-15-00181-s001.zip › clinpract-3809068-supplementary.pdf]

Supplementary Material

Table S1. Latent Constructs and Survey Items of Study Questionnaire.

Latent structure	Items	References
Perceived ease of use (PEOU)	The items account for ease of use, learning process to use mobile health apps, and the level of effort involved.<br>PEOU 1: Learning to use mobile health apps is easy for me.<br>PEOU2: No great effort is needed in using mobile health apps.	[16,19,20,54–57]
Perceived Usefulness (PU)	The items account for importance attributes related to mobile health apps.<br>PU1: Mobile health apps are beneficial for the management of my chronic condition.<br>PU2: Mobile health apps help me win time when managing my chronic condition.<br>PU3: Mobile health apps help me do things I need to do for the management of my chronic condition.<br>PU4: I find mobile health apps useful for the management of my chronic condition.	[16,19,20,54–57]
Perceived Risk (RISK)	The items relate to potential negative effects associated with the use of mobile health apps.<br>RISK1: It brings insecurity to use mobile health apps.<br>RISK2: Mobile health apps can be the cause for medical errors.<br>RISK3: Because of mobile health apps, my medical data can be used for other purposes without my approval.<br>RISK4: My medical data can be lost because of mobile health apps.	[16,24,29,42]
Digital Self-Efficacy (DSE)	The items account for the competencies in interacting with digital technologies.<br>DSE1: I know how to protect my personal data in digital environments.<br>DSE2: I can find and apply various solutions to technical problems that arise.	[20,34,58]
Perceived Cyber Insecurity (CYBER)	The items reflect fears of potential cyber security threats.<br>CYBER1: The use of mobile health apps can cause the leakage of my personal information.<br>CYBER2: My personal information can be used for other purposes when using mobile health apps.<br>CYBER3: Mobile health apps can be attacked by cyber hackers.<br>CYBER4: Mobile health apps may experience disturbances due to computer hacking.	[15,29,42]
Intention to use (INT)	The items express the intention to personally use mobile health apps and to recommendation its use to other people<br>INT1: I have the intention to use mobile health apps.<br>INT2: I will recommend the use of mobile health apps to others.	[16,19,20,54–57]

Table S2. Measurement Reliability Assessment.

A. Latent Variables Coefficients

Latent construct	Composite reliability	Cronbach's Alpha	Average Variance Extracted	Full collinearity VIFs
DSE	0.924	0.836	0.859	2.981
PEOU	0.970	0.937	0.941	3.942
PU	0.987	0.982	0.949	3.103
RISK	0.901	0.852	0.694	2.935
CYBER	0.942	0.919	0.804	2.005
INT	0.991	0.982	0.982	3.854

B. Correlations among latent variables with square roots of AVEs

	DSE	PEOU	PU	CYBER	RISK	INT
**DSE**	0.900	0.780	0.534	-0.319	-0.434	0.620
**PEOU**	0.780	0.970	0.683	-0.401	-0.551	0.715
**PU**	0.534	0.683	0.974	-0.553	-0.703	0.778
**CYBER**	-0.319	-0.401	-0.553	0.897	0.686	-0.607
**RISK**	-0.434	-0.551	-0.703	0.686	0.833	-0.732
**INT**	0.620	0.715	0.778	-0.607	-0.732	0.991

Table S3. Causality Assessment Coefficients Analysis. (a) The Warp2 bivariate causal direction ratios for the RITAM components; (b) Absolute Warp2 bivariate Causal Direction Differences; (c) The Warp3 bivariate causal direction ratios for the RITAM components; (d) Absolute Warp3 bivariate Causal Direction Differences.

	**PU**	**RISK**	**INT**
**PEOU**	0.981		1.000
**PU**		1.007	1.020
**RISK**			1.008

(a)

	**PU**	**RISK**	**INT**
**PEOU**	0.013 (n.s.*)		0.000 (n.s.)
**PU**		0.005 (n.s.)	0.016 (n.s.)
**RISK**			0.006 (n.s.)

(b)

	**PU**	**RISK**	**INT**
**PEOU**	0.979		1.000
**PU**		0.996	1.019
**RISK**			0.995

(c)

	**PU**	**RISK**	**INT**
**PEOU**	0.015 (n.s.)		0.000 (n.s.)
**PU**		0.003 (n.s.)	0.015 (n.s.)
**RISK**			0.003 (n.s.)

(d)

*p-values greater than 0.05 indicate non-significant results and are marked with 'n.s.'
